# Outbreak of Severe Vomiting in Dogs Associated with a Canine Enteric Coronavirus, United Kingdom

**DOI:** 10.3201/eid2702.202452

**Published:** 2021-02

**Authors:** Alan D. Radford, David A. Singleton, Chris Jewell, Charlotte Appleton, Barry Rowlingson, Alison C. Hale, Carmen Tamayo Cuartero, Richard Newton, Fernando Sánchez-Vizcaíno, Danielle Greenberg, Beth Brant, Eleanor G. Bentley, James P. Stewart, Shirley Smith, Sam Haldenby, P.-J. M. Noble, Gina L. Pinchbeck

**Affiliations:** Institute of Infection, Veterinary and Ecological Sciences, University of Liverpool Leahurst Campus, Neston, UK (A.D. Radford, D.A. Singleton, B. Brant, E.G. Bentley, J.P. Stewart, S. Smith, P.-J.M. Noble, G.L. Pinchbeck);; Lancaster University Centre for Health Informatics, Lancaster, UK (C. Jewell, C. Appleton, B. Rowlingson, A.C. Hale);; University of Bristol, UK (C.T. Cuartero, F. Sánchez-Vizcaíno);; Animal Health Trust, Lanwades Park, Kentford, UK (R. Newton);; The Liverpool Vets, Liverpool, UK (D. Greenberg);; Centre for Genomic Research, University of Liverpool, Liverpool (S. Haldenby)

**Keywords:** canine enteric coronavirus, dogs, enteric infections, gastrointestinal disease, outbreaks, statistical modeling, surveillance, syndromic surveillance, United Kingdom, viruses, vomiting

## Abstract

The lack of population health surveillance for companion animal populations leaves them vulnerable to the effects of novel diseases without means of early detection. We present evidence on the effectiveness of a system that enabled early detection and rapid response a canine gastroenteritis outbreak in the United Kingdom. In January 2020, prolific vomiting among dogs was sporadically reported in the United Kingdom. Electronic health records from a nationwide sentinel network of veterinary practices confirmed a significant increase in dogs with signs of gastroenteric disease. Male dogs and dogs living with other vomiting dogs were more likely to be affected. Diet and vaccination status were not associated with the disease; however, a canine enteric coronavirus was significantly associated with illness. The system we describe potentially fills a gap in surveillance in neglected populations and could provide a blueprint for other countries.

Population health data is lacking for companion animals such as dogs, cats, and rabbits, leaving a surveillance gap for endemic diseases and delayed detection of incursions of disease, such as equine influenza virus (H3N8) (*1*), avian influenza (H3N2) (*2*,*3*), and parvoviruses (*3*). In the absence of legislated programs of population surveillance, several attempts have been made to fill this gap using secondary data, particularly from pet insurance providers (*4*). More recently, researchers have exploited the rapid digitization of electronic health records (EHRs) for passive surveillance. Data can be collected at great scale and analyzed in near–real time. EHR data are now routinely used in human heath efforts (*5*–*8*), in which their timeliness, simplicity, and breadth of coverage complements surveillance based on diagnostic data (*9*,*10*). Such approaches are beginning to find healthcare value in veterinary species, especially among companion animals (*4*,*11*–*13*), a high proportion of which visit veterinarians (*14*).

In January 2020, one of the authors of this article (D.G.), a primary care veterinarian in northwest England, contacted the other authors about seeing an unusually high number of cases (≈40) of severe vomiting in dogs; responses to a social media post suggested other veterinarians might have been experiencing similar events. Vomiting is a common complaint among dogs whose owners seek treatment for them (*15*,*16*). However, documented outbreaks are rare because established vaccines are available for most common known pathogens (*17*). In the absence of robust populationwide data, such sporadic reports frequently do not raise awareness of outbreaks.

For the response we describe, we obtained data from syndromic surveillance and text mining of EHRs collected from sentinel veterinary practices and diagnostic laboratories, which we then linked with data from field epidemiology and enhanced genomic testing. In 8 weeks, using this approach, we described the temporal and spatial epidemiology, identified a possible causative agent, and provided targeted advice to control the outbreak. Ethics approval was given by Liverpool University Research Ethics Committees (Liverpool, UK; VREC922/RETH000964).

## Methods

### Data Sources

#### Veterinary Practices

During March 17, 2014–February 29, 2020, we collected data from 7,094,397 consultation records (4,685,732 from dogs and 1,846,493 from cats) from EHRs from the Small Animal Veterinary Surveillance Network (SAVSNET), a volunteer network of 301 veterinary practices (663 sites) in the United Kingdom, recruited based on convenience (*11*). In brief, EHRs included data collected during individual consultations on species, breed, sex, neuter status, age, owners’ postcodes, and vaccination status. Each EHR is also compulsorily annotated by the veterinary clinician with a main presenting complaint (MPC) at time of visit, using a questionnaire window embedded in the practice management system. Options for reasons for visit included gastroenteric, respiratory, pruritus, tumor, kidney disease, other unwell, post-op check, vaccination, or other healthy.

Given that severe vomiting was a key outbreak feature, we undertook 2 complementary analyses. First, we used regular expressions to identify clinical narratives describing frequent vomiting, but excluded common false positive search results (Appendix Table 1). Second, we used data on prescriptions to describe the frequency of all veterinary-authorized products containing the antiemetic maropitant (*18*). We calculated trend lines using Bayesian binomial generalized linear modeling trained on weekly prevalence during 2014–2019 (*19*), which allowed us to identify extreme (>99% credible interval [CrI]) or moderate (>95% CrI) observations.

### Laboratories

SAVSNET also collects EHRs from participating diagnostic laboratories on samples submitted from more than half of UK veterinary practices. Canine diagnostic test results from January 2017 through February 2020 were queried from 6 laboratories for 6 gastroenteric pathogens. Test numbers, percentage of positive results, and associated 95% CIs were summarized (Table 1). The number of sites was surmised from the submitting practices’ postcodes.

**Table 1:** Results of laboratory diagnostic tests for pathogens associated with gastroenteric disease in dogs for samples collected during January 2017–February 2020, United Kingdom*

Pathogen	Method	No. tests	No. laboratories†	Unique sites‡	% Positive (95% CI)	Peak month, % positive (95% CI)
CeCoV	PCR	5,167	4	839	20.69 (19.58–21.79)	2020 Feb, 34.8 (27.81–41.85)
Canineparvovirus	PCR	5,499	6	965	6.62 (5.96–7.28)	2017 Nov, 13.28 (7.38–19.18)
Giardia	PCR	5,636	6	894	23.78 (22.66–24.89)	2018 Jan, 33.96 (26.58–41.35)
*Salmonella* spp.	Culture	114,722	6	2,951	0.87 (0.81–0.92)	2018 Nov, 1.28 (0.87–1.70)
*Campylobacter* spp.	Selective culture	111,983	6	2,947	16.10 (15.88–16.31)	2017 Dec, 23.02 (21.44–24.60)
*Clostridium perfringens*	Enterotoxin PCR	5,138	3	2,947	16.10 (15.88–16.31)	2017 Dec, 23.02 (21.44–24.60)

*CeCoV, canine enteric coronavirus. †Number of diagnostic laboratories contributing test results. ‡Number of unique veterinary practices sites submitting samples to the laboratories.

### Questionnaires

Online questionnaires to enable case reporting were made available to both veterinarians and owners beginning January 29, 2020. The required case definition of >5 vomiting episodes in a 12-hour period was based on clinical observations of early cases. Veterinarians were also asked to complete control questionnaires. Initially, we requested only controls matched to veterinary practices contributing case data; however, to increase recruitment, a nonmatched control questionnaire open to any veterinarian was deployed on February 5. The questionnaires (Appendix) requested a range of information including owner postcode, animal signalment, vaccination status, clinical signs, treatment and diagnostic testing, animal contacts, diet, and recovery status.

We performed all statistical analyses using R version 3.6.1 (https://cran.r-project.org). Case details were described for both veterinarian- and owner-reported data. We calculated proportions and 95% CIs for categorical variables and median and range for continuous variables. We constructed univariable and multivariable mixed-effects logistic regression models using data submitted by veterinarians using R package lme4. Explanatory variables from univariable logistic regression were considered in multivariable models for likelihood ratios of p≤0.20, which underwent manual stepwise backward elimination to reduce Akaike’s and Bayesian information criteria. Practice was included as a random effect. We assessed confounding by the effect on model fit with sequential removal of variables and assessed 2-way interaction terms for improved model fit. We defined final statistical significance as p <0.05.

### Spatiotemporal Analysis of Cases

We obtained records of consults weekly during November 4, 2019–March 21, 2020; cases were geolocated by pet owners’ postcodes. We considered records of gastroenteric MPC as a binary outcome (i.e., 1 for gastroenteric consult, 0 for nongastroenteric consult). We used a logistic geostatistical model to investigate spatial clustering of cases for each week. We defined a spatial hotspot as a location having 95% posterior probability of prevalence exceeding the national mean prevalence over any 1-week period. With no discernible epidemic wave apparent over successive weeks, we aggregated weekly measures across the study period to show the number of weeks each location was a hotspot (Appendix).

### Sample Collection, PCR, and Phylogenetic Analyses

Veterinarians submitting questionnaires were also asked to submit samples for microbiological testing including mouth swabs, fecal samples, and for gastrointestinal cases, vomit. In brief, we extracted nucleic acids using a QIAGEN QIAamp viral RNA kit (https://www.qiagen.com), reverse transcribed samples using ThermoFisher Superscript III (https://www.thermofisher.com), and tested for canine enteric coronavirus (CeCoV) by M-gene PCR (*20*). To expedite results and reduce contamination risks, the PCR was run as a single-stage PCR rather than as the published nested reaction. We purified positive samples using QIAquick (QIAGEN) and sequenced them bidirectionally (Sanger sequencing; Source Biosciences, https://www.sourcebioscience.com) to produce consensus sequences (ChromasPro 2.1.8, http://technelysium.com.au).

To rapidly explore the potential involvement of other viruses, we extracted nucleic acid from 19 random cases and 5 controls for deep sequencing. RNA was amplified by sequence-independent, single-primer-amplification (*21*), multiplexed libraries were prepared using 30 ng of cDNA with an Oxford Nanopore SQK-LSK109 ligation sequencing kit (Oxford Nanopore, https://nanoporetech.com) and sequenced using an Oxford Nanopore MinION Mk1B device for 48 hours. To perform real-time fast basecalling, we used the Oxford Nanopore MinKNOW Guppy toolkit and FASTQ files uploaded to an Oxford Nanopore EPI2ME data analysis platform for identification.

For deeper sequencing coverage, we also processed 10 samples (6 CeCoV-positive cases, 3 negative cases, 1 control) for Illumina sequencing at the University of Liverpool Centre for Genomic Research (https://www.liverpool.ac.uk/genomic-research). We treated nucleic acids with RNase and prepared fragment libraries using a NEBNext Ultra II kit (https://www.neb.com) before performing paired-end, 2 × 150–bp sequencing on an Illumina HiSeq 4000 system (https://www.illumina.com). Adaptor sequences were trimmed using cutadapt (https://cutadapt.readthedocs.io) and sickle (https://github.com), with a minimum quality score of 20. Reads >19 bp matching the dog genome (CanFam3.1, http://genome.ucsc.edu) using Bowtie2 sequence alignment tool (http://bowtie-bio.sourceforge.net) were removed. Remaining reads were assembled using the SPAdes toolkit (https://github.com) and contigs >700 nt blasted against the NCBI RefSeq nonredundant proteins database (https://www.ncbi.nlm.nih.gov/refseq). Sequences matching CeCoV were aligned using the ClustalW multiple sequence alignment program (https://www.genome.jp) and phylogenies reconstructed using bootstrap analyses and neighbor-joining in MEGA6 software (https://www.megasoftware.net). Each sequence was assigned a local laboratory number based on the order in which the sequences were analyzed.

## Results

### Syndromic Surveillance

**Figure 1:**
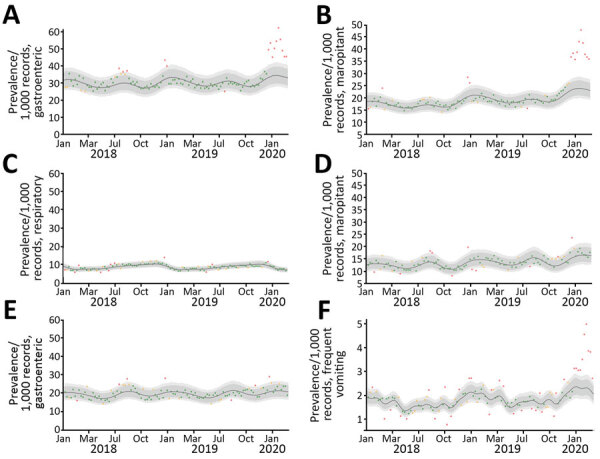
Observed prevalence of main presenting complaint (MPC) and maropitant use in cats and dogs, per 1,000 consultations, in investigation of dogs with vomiting, United Kingdom, January 2017–February 2020. A) Canine records labeled as gastroenteric MPC; B) canine records in which maropitant was prescribed; C) canine records labeled as respiratory MPC; D) feline records in which maropitant was prescribed; E) feline records labeled as gastroenteric MPC; and F) frequent vomiting in dogs based on regular expression searches of the clinical narratives. Red points represent the extreme outliers (outside the 99% credible interval [CrI]), orange points the moderate outliers (outside the 95% CrI, but within the 99% CrI), and green points the average trend (within the 95% CrI).

On the basis of MPCs identified in the EHRs, we found a specific and significant increase in the number of dogs recorded as exhibiting gastroenteric signs; the final 10 weeks, during December 2019–March 2020, were outside the 99% CrI (extreme outliers; Figure 1, panel A). A similar trend was observed in maropitant therapy for dogs (Figure 1, panel B). Both measures, peaked in the week ending February 2, 2020, at approximately double the preceding baseline. We observed no similar trends for respiratory disease in dogs, for gastroenteric MPCs, for maropitant treatment in cats (Figure 1, panels C–E), or for antibiotic use in dogs (data not shown), together suggesting the signal was specific to canine gastroenteric disease, a finding supported by similar increases in the regular expression identifying vomiting dogs (Figure 1, panel F).

**Figure 2:**
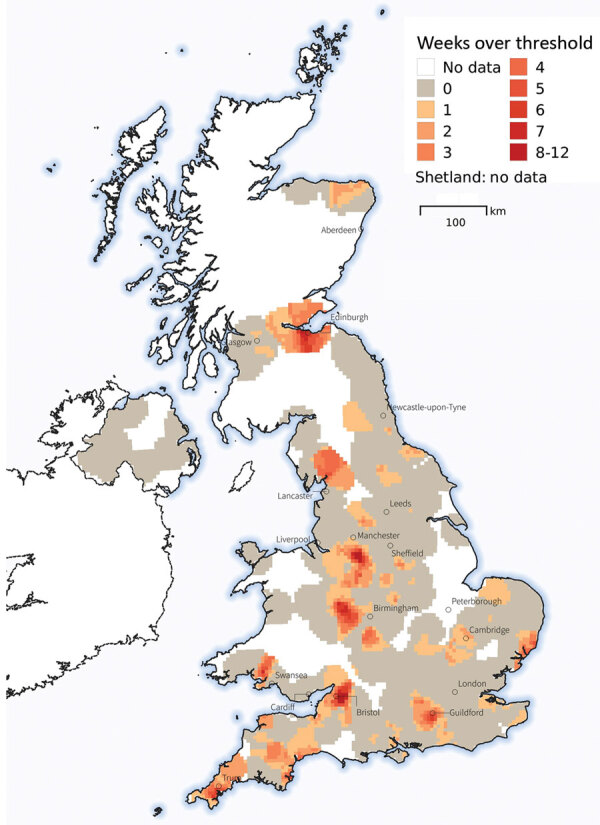
Rates of gastroenteric veterinary consults for dogs during November 4, 2019–March 21, 2020, in investigation of dogs with vomiting, United Kingdom. Consults were geolocated to owners’ postcodes, with gastroenteric main presenting complaint as a binary outcome (1 for gastroenteric consult, 0 for a nongastroenteric consult). Colored areas represent the number of weeks a given location had a 95% posterior probability of prevalence exceeding the national mean prevalence in any week. The geostatistical modeling approach used is further detailed in the Appendix.

Spatiotemporal mapping of weekly cases of gastroenteric MPC showed prevalence was spatially clustered (Figure 2). In particular, locations in northwest and southwest England and in Edinburgh, Scotland, had strong evidence of many weeks of prevalence higher than the national mean.

### Diagnostic Tests

**Figure 3:**
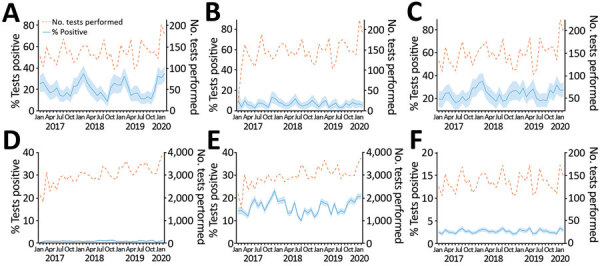
Diagnostic test findings during January 2017–February 2020 in investigation of dogs with vomiting, United Kingdom. A) Canine enteric coronavirus PCR; B) canine parvovirus PCR; C) *Giardia* PCR; D) *Salmonella* spp. selective culture; E) *Campylobacter* spp. selective culture; F) *Clostridium perfringens* enterotoxin PCR results. Blue shading represents 95% CI.

The patterns of test results for different PCR tests, generally carried out concurrently, were broadly similar (Figure 3, panels A–C). The same was true for results based on cultured samples (Figure 3, panels D, E). Of particular interest, CeCoV showed strong seasonality, positive tests peaking during the winter months (Figure 3, panel A). However, similar peaks seen in previous years suggested the observed peak in February 2020 could not itself explain this outbreak.

### Questionnaire

By March 1, 2020, a total of 1,258 case questionnaires had been received. After excluding 59 questionnaires missing key data, we used data from 165 veterinary-reported cases, 1,034 owner-reported cases (Table 2), and 60 veterinary-reported controls (Appendix Table 2) for analyses.

**Table 2:** Veterinary- and owner-reported case questionnaire responses pertaining to signalment, health history, contacts, and feeding habits among dogs with vomiting, United Kingdom, January 2017–February 2020*

Question	Veterinarian-reported cases, n = 165			Owner-reported cases, n = 1,034	
	% Responses (95% CI)	No. unknown		% Responses (95% CI)	No. unknown
Veterinary practice location					
England	80.6 (74.6–86.7)	NA		89.8 (87.9–91.6)	NA
Wales	12.1 (7.1–17.1)	NA		4.5 (3.2–5.7)	NA
Scotland	4.9 (1.6–8.1)	NA		4.5 (3.2–5.7)	NA
North Ireland	1.2 (0.0–2.9)	NA		1.1 (0.4–1.7)	NA
Republic of Ireland	1.2 (0.0–2.9)	NA		0.1 (0.0–0.3)	NA
Isle of Man	0	NA		0.2 (0.0–0.5)	NA
Sex					
F	42.4 (34.9-50.0)	NA		43.7 (40.7-46.7)	NA
M	57.6 (50.0–65.1)	NA		56.3 (53.3–59.3)	NA
Neutered‡	69.1 (62.0–76.2)	NA		70.1 (67.3–72.9)	NA
Intact‡	30.9 (23.8-37.9)	NA		29.9 (27.1-32.7)	NA
Vaccinated within past 3 y†	94.6 (91.1–98.0)	NA		88.4 (86.5–90.4	13
Distemper	92.7 (88.8–96.7)	NA		49.7 (46.7–52.8)	NA
Infectious hepatitis	92.1 (88.0–96.2)	NA		40.4 (37.4–43.4)	NA
Parvo	92.1 (88.0–96.2)	NA		55.4 (52.4–58.5)	NA
Parainfluenza	53.9 (46.3–61.6)	NA		37.4 (34.5–40.4)	NA
Leptospirosis	92.7 (88.8–96.7)	NA		49.2 (46.2–52.3)	NA
Kennel cough	46.7 (39.0–54.3)	NA		40.4 (37.4–43.4)	NA
Rabies	2.4 (0.1–4.8)	NA		1.3 (0.6–1.9)	NA
Herpes	0.6 (0.0–1.8)	NA		NA	NA
Dewormed within past 3 mo	86.2 (80.5–92.0)	27		69.8 (67.0–72.7)	50
Lives in multidog household	34.6 (27.3–41.8)	NA		47.4 (44.3–50.4)	NA
>1 dogs in household vomited	54.4 (41.3–67.4)	NA		55.9 (51.5–60.3)	NA
Regular contact with other species†	54.9 (46.1–63.8)	43		44.1 (41.1–47.1)	NA
Cats	64.2 (52.6–75.8)	NA		62.3 (57.8–66.7)	NA
Horses	20.9 (11.1–30.7)	NA		28.3 (24.2–32.4)	NA
Cattle or sheep or both	25.4 (14.9–35.9)	NA		22.2 (18.3–26.0)	NA
Pigs	3.0 (0.0–7.1)	NA		1.5 (0.4–2.7)	NA
Poultry	13.4 (5.2–21.7)	NA		14.0 (10.8–17.2)	NA
Rabbits	7.5 (1.1–13.8)	NA		5.7 (3.6–7.8)	NA
Other species	11.9 (4.1–19.8)	NA		20.6 (16.9–24.3)	NA
Contact with other vomiting species	13.5 (7.1–19.9)	54		17.4 (14.6–20.2)	320
Recent travel history†	31.4 (23.0–39.8)	47		26.7 (24.0–29.4)	NA
Boarding kennel	8.1 (0.0–17.0)	NA		9.1 (5.7–12.5)	NA
Group training/behavior classes	24.3 (10.3–38.3)	NA		35.5 (29.9–41.2)	NA
Doggie day care facility	48.7 (32.3–65.0)	NA		39.5 (33.7–45.3)	NA
Overseas	2.7 (0.0–8.0)	NA		0.7 (0.0–1.7)	NA
Rescue kennel	0.0 (0.0–0.0)	NA		0.4 (0.0–1.1)	NA
Other	18.9 (6.1–31.7)	NA		20.3 (15.5–25.0)	NA
Provided known food type†	95.2 (91.9–98.4)	8		100.0 (100.0–100.0)	NA
Proprietary dog food	95.5 (92.3–98.8)	NA		85.9 (83.8–88.0)	NA
Home-cooked diet	6.4 (2.5–10.2)	NA		10.4 (8.6–12.3)	NA
Raw meat	5.1 (1.6–8.6)	NA		15.9 (13.6–18.1)	NA
Table scraps	14.7 (9.1–20.2)	NA		16.1 (13.8–18.3)	NA
Scavenged food	36.6 (28.7–44.4)	20		19.9 (17.4–22.4)	24

*NA, not available. ‡Includes both female and male animals. †Multiple responses for the same dog are possible.

Most cases were from households in England (Table 2). Median case age at examination was 4.0 years (range 0.3–15.0 years) based on veterinary reports and 4.8 years (range 0.2–15.5 years) based on owner reports. Most animals had been vaccinated against core pathogens (*17*) and leptospirosis within the preceding 3 years and dewormed within the previous 3 months. A range of breeds (data not presented) were observed, broadly corresponding to previous studies (*6*). Most cases were fed dog food, with ≈20%–37% of dogs scavenged food when walked. Of those from multidog households, just over half reported the presence of another dog recently vomiting within the same household. Around 30% of dogs had recently traveled, most commonly visiting a daycare facility.

Date of onset of clinical signs ranged from November 16, 2019, through February 28, 2020, for veterinary-reported cases, and September 4, 2019, through March 1, 2020, for owner-reported cases. Most cases involved inappetence (75.6%–86.1%) and vomiting without blood (88.7%–91.5%) (Table 3). Approximately half of cases reported diarrhea, most without blood. Diagnostic testing was performed in 32.1% of veterinary-reported cases, most (78.9%) using hematology or biochemistry assays, or both.

**Table 3:** Veterinarian reported and owner-reported case questionnaire responses pertaining to clinical signs, diagnostic and management strategies, and case recovery likelihood and time among dogs with vomiting, United Kingdom, January 2017–February 2020*

Question	Veterinarian-reported cases, n = 165			Owner-reported cases, n = 1,034	
	% Responses (95% CI)	No. unknown		% Responses (95% CI)	No. unknown
Clinical signs					
Vomiting without blood	91.5 (87.3–95.8)	NA		88.7 (86.8–90.6)	NA
Vomiting with blood	8.5 (4.2–12.8)	NA		11.3 (9.4–13.3)	NA
Diarrhea without blood	37.0 (29.6–44.4)	NA		46.2 (43.2–49.3)	NA
Diarrhea with blood	10.9 (6.1–15.7)	NA		12.3 (10.3–14.3)	NA
Melaena	1.8 (0.0–3.9)	NA		NA	NA
Pyrexia	12.7 (7.6–17.8)	NA		15.4 (13.2–17.6)	NA
Inappetence	86.1 (80.8–91.4)	NA		75.6 (73.0–78.3)	NA
Weight loss	18.2 (12.3–24.1)	NA		34.9 (32.0–37.8)	NA
Lethargy	9.1 (4.7–13.5)	NA		6.3 (4.8–7.8)	NA
Diagnostic testing performed	32.1 (25.0–39.3)	NA		18.3 (15.9–20.7)	NA
Treatment provided to dog	92.1 (88.0–96.2)	NA		61.7 (58.7–64.7	13
Recovery status known	88.5 (83.6–93.4)	19		98.4 (97.6–99.1)	17
Recovery <24 h	5.5 (2.0–8.9)	NA		2.9 (1.8–3.9)	NA
Recovery in 24–48 h	17.6 (11.8–23.4)	NA		21.1 (18.6–23.7)	NA
Recovery in 3–7 d	30.9 (23.8–38.0)	NA		36.2 (33.2–39.1)	NA
Recovery in 7–14 d	2.4 (0.1–4.8)	NA		5.9 (4.5–7.4)	NA
Recovery in over 14 d	2.4 (0.1–4.8)	NA		2.1 (1.2–2.9)	NA
Dog currently vomiting	7.9 (3.8–12.0)	NA		9.4 (7.6–11.2)	NA
Dog not vomiting but still unwell	21.2 (15.0–27.5)	NA		21.4 (18.9–24.0)	NA
Dog died	0.6 (0.0–1.8)	NA		1.0 (0.4–1.6)	NA

*NA, not available.

Dogs in >90% of veterinary-reported cases were treated, compared with in 61.7% of owner-reported cases. In both, antiemetics were most often prescribed: in 89.1% (CrI 84.3%–93.9%) of veterinary-reported cases and in 48.1% (CrI 45.0%–51.1%) of owner-reported cases. The most common recovery time was 3–7 days; the dogs died in 0.6% of veterinary-reported and 1.0% of owner-reported cases.

Descriptive data about the control population, submitted by veterinarians, and univariable findings from analyses of the veterinary case controls are presented in Appendix Tables 2 and 3; multivariable findings are shown in Table 4. Both neutered and nonneutered male dogs were at significantly increased odds of contracting the illness, compared with neutered females, as were dogs living in the same household as another dog that had also been vomiting compared to those in households where other dogs were healthy. However, dogs living in a single-dog household were at increased odds of contracting the illness compared with dogs living in the same household as another dog that had not recently vomited. Dogs that had been in recent contact with another animal species (including humans) that had recently vomited were at reduced odds of vomiting, compared with those who had not. Other potential causes considered early in the outbreak, including foodborne etiologies, vaccine preventable diseases, or the possibility of interspecies transmission, were not significantly associated (Appendix Table 3).

**Table 4:** Mixed effects multivariable logistic regression model investigating odds of being a veterinarian-reported prolific vomiting case among 165 cases and 60 controls in investigation of dogs with vomiting, United Kingdom, January 2017–February 2020*

Variable	β	SE	OR (95% CI)	p value†
Intercept	–0.36	0.42	NA	NA
F, neutered	NA	NA	Referent	NA
F, intact	0.77	0.55	2.15 (0.74–6.26)	0.16
M, neutered	0.81	0.40	2.25 (1.03–4.91)	0.04
M, intact	1.34	0.59	3.82 (1.20–12.15)	0.02
Multidog household, no other dogs vomiting in the same household	NA	NA	Referent	NA
Multidog household, other dogs vomiting in the same household	1.15	0.53	3.16 (1.11–8.97)	0.03
Single-dog household	1.17	0.40	3.23 (1.47–7.11)	<0.01
No contact with other species vomiting	NA	NA	Referent	NA
Confirmed contact with other species vomiting	–1.23	0.48	0.29 (0.12–0.74)	0.01
Unknown contact with vomiting other species	0.63	0.42	1.88 (0.83–4.26)	0.13

*β, β-value (coefficient). †p value <0.05 indicates significant findings.

### Sampling and Molecular Testing

During January 30–March 12, 2020, we collected a total of 95 samples from 71 animals (50 cases, 21 controls): 22 from feces, 60 from oral swabs, and 13 from vomit. Dogs with prolific vomiting were significantly more likely to test positive for CeCoV in >1 sample (17/50, 34%) compared with controls (0/21) (p = 0.002 by Fisher exact test). Positive test results were most likely in samples from feces (10/16 [62.5%] cases, 0/6 controls; p = 0.01) and vomit (6/13 [46%] cases, 0 controls). Samples from oral swabs were least likely to test positive (7/43 [16%] cases, 0/17 controls; p = 0.17). Of 17 CeCoV-positive cases, 12 met the case definition, 2 did not (<5 episodes of vomiting in 12 hours), and 3 lacked questionnaire data.

**Figure 4:**
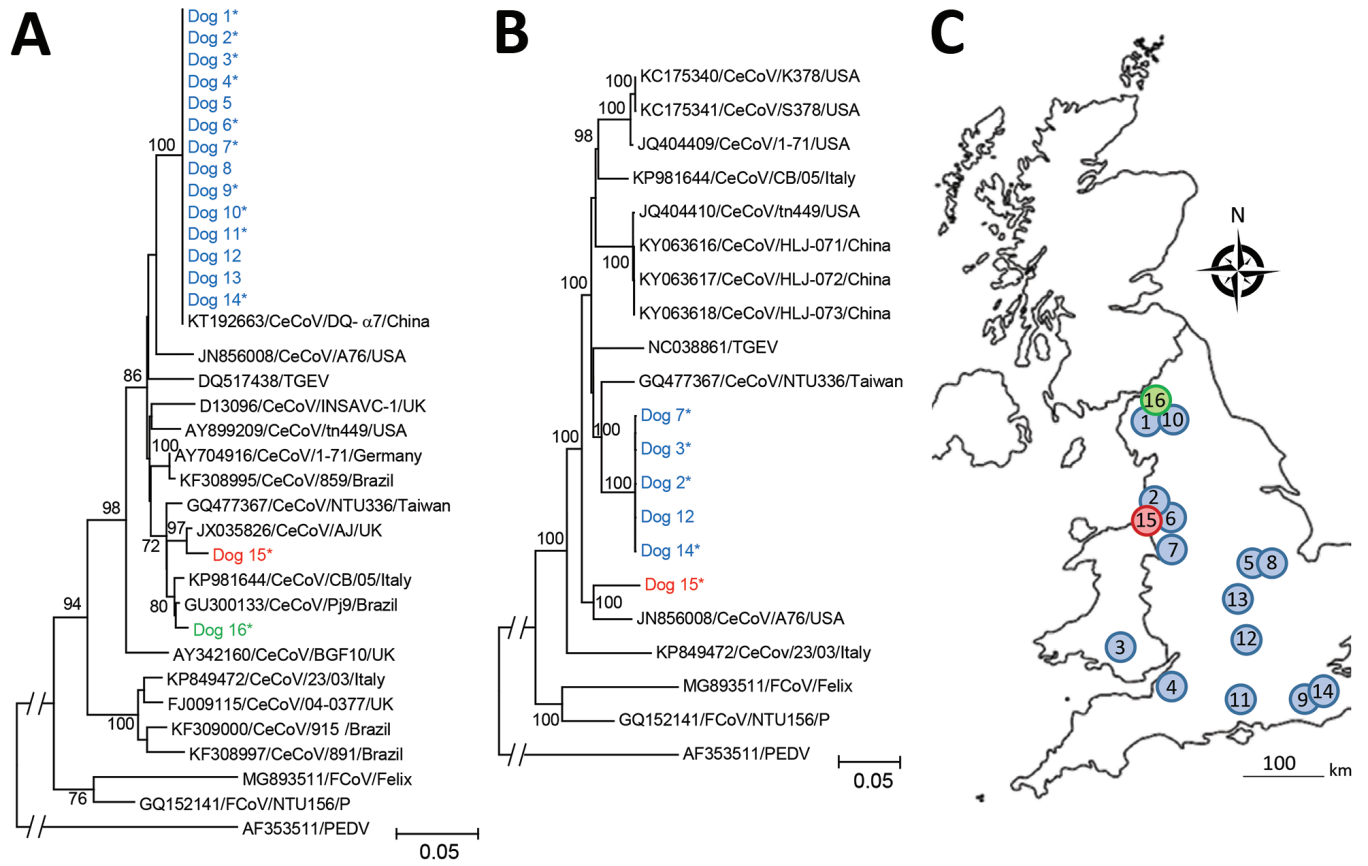
Phylogenetic analysis of canine enteric coronavirus strains, including locations were sequences were obtained, in investigation of dogs with vomiting, United Kingdom. Trees are based on nucleotide sequences for M-gene (final alignment 299 positions) (A) and whole genome (final alignment 26,564 positions) (B). Evolutionary analysis was performed using the neighbor-joining method. Bootstrap testing using 1,000 replicates was applied; only values >70 are indicated. Sequences identified in this study are indicated in blue (strain 1), red (strain 2), and green (strain 3). Asterisks (*) indicate samples from animals meeting the case definition. Each phylogeny included closest matches in GenBank, as well as representative published canine coronavirus, feline coronavirus, and transmissible gastroenteritis virus isolates. Scale bars indicate substitutions per site. C) Approximate geographic location of sequences obtained in this study, number- and color-matched to sequences shown in panels A and B.

We gathered useable M-gene sequences from 21 samples (16 dogs). When we sequenced 2 samples from the same animal, the sequences were identical and subsequently represented only once in analyses (Figure 4). All sequences clustered with previously reported type II CeCoVs (*22*) in 1 of 3 lineages. Sequences from 14 of 16 dogs were identical, suggesting a single outbreak strain geographically distributed across England. Sequences from dogs 15 and 16 were phylogenetically distinct.

Results of MinION sequencing rapidly confirmed an alphacoronavirus as the predominant virus (24,190 out of 33,826,933 reads) and failed to identify any other prevalent candidates (next highest, betabaculovirus: 4,541 reads). Although bacterial reads were present in high numbers, none showed consistently high results across most samples.

Complete CeCoV genomes were assembled from 6 PCR-positive cases by Illumina sequencing. We identified no coronavirus sequences in 3 cases and 1 control that tested negative for CeCoV by PCR. The only other mammalian virus sequence detected matched a canine rotavirus (1 case, 1 control; data not presented). Consistent with M-gene sequencing, 5 of the CeCoV genomes clustered together (>99% similarity), distinct from the genome from dog 15 (Figure 4). The outbreak strain was most similar to a virus from Taiwan isolated in 2008 from a young dog with diarrhea (94.5% similarity; L. Chueh, pers. comm. [email] Apr. 27, 2020) and did not show any obvious sequence differences to published strains that might explain the unusual pattern of disease observed in the outbreak. Based on spike gene analyses, the outbreak strain clustered with IIb, having a TGEV-like N-terminal spike domain (*23*). Sequences were submitted to GenBank (accession nos. MT877072, MT906864, and MT906865).

## Discussion

Using EHRs annotated with syndromic information by veterinarians, we rapidly identified an outbreak of canine gastroenteric disease that had started in November 2019. This finding was corroborated by parallel increases in relevant prescriptions and records of frequent vomiting. These data were augmented by data from responses to a questionnaire, diagnostic laboratories, and enhanced microbiological analyses. This system enabled us to determine case definitions and outcomes and to identify risk factors as well as a potential viral cause, within a 3-month period; findings were rapidly disseminated to veterinarians (*24*,*25*) and owners. This combined approach represents an efficient system that can fill a previously neglected national population health surveillance need for companion animals.

The first indication of an outbreak came from time-series analyses of syndromic data. Such syndromic surveillance is increasingly being used to monitor the impact of national events like natural disasters and bioterrorism on human population health, as well as changes in gastroenteric and influenza-like illness (*6*–*9*). Such data can be simple to collect, provide real-time wide geographic coverage, and be flexibly applied to different conditions (*10*,*11*). Although in some cases these data can identify outbreaks earlier than more active surveillance, their predictive value can sometimes be low, particularly where there is a low signal to noise complaint ratio. In our case, the outbreak was large compared with background levels, associated with near doubling of the gastroenteric syndrome, and had many weeks in which the syndrome statistically exceeded the baseline.

The richness of data within EHRs enabled us to validate this outbreak using numbers of antiemetic prescriptions and text mining. Prescription data have been used to understand, for example, human health inequalities (*26*), and the use of critical antimicrobials in both humans (*27*) and animals (*28*,*29*). We used these data to identify and track an outbreak, benefitting from a clear link between the syndrome (vomiting) and its therapy (antiemetic). It will be useful to identify other disease-therapy associations that could be used for similar surveillance.

We used text mining to identify records of frequent vomiting in clinical narratives. Such approaches can circumvent the need for practitioner-derived annotation and be flexibly and rapidly adapted to emerging syndromes as soon as case-definitions are determined. Similar approaches have been described in human health for conditions such as fever (*30*–*32*) but can suffer low sensitivity (*31*). Indeed, the outbreak peak based on text mining was ≈20% of that based on MPC analysis. However, it is also likely the outbreak as defined by the MPC included a considerable number of animals with milder signs that would not be detected by data mining using the regular expression developed here. Although data from text mining are unlikely to give an accurate estimate of the true prevalence of a given condition, they can still be used to track outbreaks.

To compliment syndromic surveillance, we implemented a rapid case-control study, collecting >1,200 responses from veterinarians and owners in 4.5 weeks. There was no evidence for similar disease in people or other species. The timing of the outbreak as shown by case data was in broad agreement with our syndromic surveillance. Questionnaires from owners and veterinarians were in broad agreement on date of onset, geographic density, clinical signs, and recovery. These data informed targeted health messages posted online and on social media on February 28, 2020, 4 weeks after we first became aware of the outbreak.

Clearly, evidence of transmission driving the outbreak was vital to providing disease control advice. Dogs in multidog households were more likely to vomit if other dogs in the household were also affected, suggesting either transmission between dogs or a common environmental source; these observations informed advice to the public around isolating affected dogs. Of note, dogs in single-dog households were also at increased odds of being affected compared to multidog households where only a single dog was vomiting. Some authors have shown that dogs from single-dog households are walked more and therefore could be at greater risk for infection (*33*). Factors affecting dog walking are clearly likely to be important for control of infectious disease transmission and should be explored further.

In addition to collecting epidemiologic data, we collected microbiological samples from cases and controls. Based on its known (*34*) and observed seasonality (Figure 3, panel A), we tested all samples for CeCoV. Cases were significantly more likely to show positive results both when all samples (oral swabs, feces and vomit) were considered or when just fecal samples were considered, suggesting a possible role for CeCoV in the outbreak. However, many case samples tested negative: 33 of 50 overall, 6 of 16 dogs for which feces samples were submitted, and 7 of 13 dogs for which vomit samples were submitted. There are several potential reasons for these negative findings, including the sensitivity of the PCR, the high numbers of oral swabs (although simpler to collect, oral swabs were more likely to test negative), the timing of samples in relation to viral shedding, and the storage and transport of samples. In addition, it is important to note that our case definition, based as it was on a syndrome and lacking more specific confirmatory testing, is likely to include some animals that were not part of the outbreak. Indeed, at its peak, the outbreak only doubled the background level of gastroenteric disease seen at other times of the year; therefore, we might expect only half of our cases to be truly associated with the outbreak.

Sequencing results identified a predominant CeCoV strain in outbreak cases across the United Kingdom, in contrast with earlier studies showing that CeCoV strains tend to cluster in households, veterinary practices, or local areas (*35*). This finding lends further support to the role of this strain in the observed outbreak. In Sweden, a single strain was also implicated in several small wintertime canine vomiting outbreaks (*36*); genetically, however, the virus strain we identified was distinct from the strain from Sweden (data not shown). Ultimately, it will be necessary to perform a challenge study to confirm or refute the role of this CeCoV strain as the cause of this outbreak, as well as to explore the range of clinical signs associated with infection.

If this strain is proven to be the cause of the outbreak, several features mark the observed pattern of disease as unusual, including the outbreak scale, its geographic distribution, the severity of signs in some animals, a lack of notable viral co-infections, and the involvement of adult dogs. CeCoV is generally associated with mild gastroenteritis (*37*). Although sporadic outbreaks of more severe hemorrhagic diseases with high mortality (*38*–*40*), as well as systemic diseases (*41*,*42*), have been reported, these typically affect individual households, and are often associated with mixed infections (*43*). Such observations suggest that the genetic variability of CeCoVs may affect virulence and are supported by experimental infections recreating more severe disease (*38*). The genetic mechanism underlying such shifts in virulence in CeCoV have not been defined. However, mutations impacting virulence are described in closely related alphacoronaviruses (*44*–*47*).

In conclusion, this multidisciplinary approach enabled a rapid response to a newly described outbreak of canine gastroenteritis and identified a CeCoV as a potential cause. Previous CeCoV seasonality suggests further outbreaks may occur. Having such an efficient surveillance system provides the ideal platform to inform and target population health messaging. Several challenges remain for addressing the lack of national population health structures for companion animals: to systematically capture discussions of disease in social and mainstream media; to sustainably fund these activities, which currently are largely resourced by research grants; to understand and broaden the representativeness of such sentinel networks; and to link surveillance information with agencies empowered to act (*12*).

Appendix Additional details of methodology for geospatial modelling and questionnaires used to collect case and control data from veterinarians and owners.
