# Supplementary material for: Outbreak of Severe Vomiting in Dogs Associated with a Canine Enteric Coronavirus, United Kingdom
Source: Emerg Infect Dis. 2021 Feb;27(2):517–28. doi: 10.3201/eid2702.202452 (PMC7853541; doi:10.3201/eid2702.202452)
Supplement: Appendix — Additional details of methodology for geospatial modelling and questionnaires used to collect case and control data from veterinarians and owners. [file 20-2452-Techapp-s1.pdf]

Article DOI: https://doi.org/10.3201/eid2702.202452

Nationwide Outbreak of Severe Vomiting in Dogs Associated with a Canine Enteric Coronavirus, United Kingdom

Appendix

Supplementary Information on Geostatistical Modelling

The geostatistical model used to investigate spatial clustering for severe vomiting in dogs makes use of owner-geolocated prevalence data based on total consults recorded in SAVSNet. Below, we first describe the geostatistical model setup, before describing how the results were presented using geographical information systems methods.

Geostatistical Model for Prevalence

For each week between 4th November 2019 and 21st March 2020, our data comprise an indicator $y_i \in \{0,1\}$ for $i = 1, \dots, n_t$ consults recorded. For each consult, we additionally have the centroid of the owner's postcode area x_i in Cartesian coordinates (OSGB 1936 coordinate system).

We model y_i as a Bernoulli random variable such that

$$y_i \approx \text{Bernoulli}(p_i)$$

with

$$\text{logit}(p_i t) = \alpha + S(x_i).$$

$S(x)$ is a spatial Gaussian process such that

$$S(x) \approx \text{MultivariateNormal}(0, \Sigma^2)$$

Σ^2 is a covariance matrix defined by a Matérn correlation function:

$$\Sigma_{ii}^2 = \sigma^2 \Sigma_{ij}^2 = \sigma^2 \left(1 + \frac{\sqrt{3\|x_i - x_j\|^2}}{\phi}\right) exp\left[-\frac{\sqrt{3\|x_i - x_j\|^2}}{\phi}\right]$$

where $\|x_i - x_j\|$ is the Euclidean distance between locations x_i and x_j, σ^2 is the sill variance of the spatial Gaussian process, and ϕ is the length scale (*1*).

The computation of the log posterior probability density for this model involves the inversion of Σ^2 which becomes computationally prohibitive beyond a few hundred points. Since in a typical week $n \approx 24000$, we use the inducing point approximation of Banerjee et al. (*2*). Here, we choose a set of m knot points $x_i^\star, i = 1,\ldots,m$ and let

$S(x) \approx \Sigma_{xx^\star}^2 \left(\Sigma_{x^\star x^\star}^2\right)^{-1} s^\star$ (1)

where $s^\star$ is a realisation of the Gaussian process at knots $x^\star$. In practice, we find that 300 knot points positioned using K-means clustering on x gives satisfactory computational performance with negligible information loss compared to 600 and 900 knot points positioned similarly.

Finally, we investigated the requirement for a “nugget”, or uncorrelated, random effect by adding a variance component to the diagonal of Σ^2, i.e. $\Sigma_{ii}^2 = \sigma^2 + \tau^2$. However, this did not improve the model fit and was removed for the sake of parsimony.

This model was fitted to the consulting data in a Bayesian framework. The following prior distributions were chosen to reflect relative *a priori* ignorance about parameters:

$$\begin{array}{ccc} \alpha & \sim & \text{Normal}(0,100) \\ \phi & \sim & \text{Gamma}(2,0.1) \\ \sigma_s & \sim & \text{Gamma}(1,1) \end{array}$$

The No-U-Turn Sampling (NUTS) Markov-chain Monte Carlo method was used to draw samples from the joint posterior distribution $\pi(\alpha, \phi, \sigma^2, s(x)|x, y)$, and implemented in Python v3.6 using the PyMC3 v3.8 embedded probabilistic programming language. Source code is available at https://github.com/SAVSNET.

GIS Presentation of Results

Using Equation (1), the posterior samples of $S(x^\star)$ were projected onto a 5km resolution grid of points z within the outline of the UK (*3*). This gave a numerical approximation of the

predictive distribution $\pi(S(z)|y,x)$ of the posterior log odds ratio for a consult being for severe vomiting, relative to the national-level odds (i.e. $\hat{\alpha}$). These results were summarised by calculating the probability that $z_i > 0$ (or equivalently $e^z > 1$) for all grid locations.

The model was run for all weekly intervals $t = 1, \ldots, T$ between 4th November 2019 and 21st March 2020. In the absence of a strong wave-like progression of disease throughout the UK, the results were summarized as

$$\omega_k = \sum_{t=1}^{T} [Pr(z_i > 0|y,x)] \geq 0.95$$

for all grid points k. In other words, ω_k represents the number of weeks where a particular grid point k was predicted to have a positive case odds ratio above 1 with a posterior probability of at least 0.95 compared to the national average prevalence in each week. It therefore provides an estimate of locations that were at higher risk of positive cases compared to the national average over time during the outbreak.

All calculations were performed in Python v3.6, and cartography was performed in QGIS v3.12.

References

<bok>1. Diggle P, Ribeiro PJ. Model-based geostatistics. New York: Springer. 2007.</bok>

<jrn>2. Banerjee S, Gelfand AE, Finley AO, Sang H. Gaussian predictive process models for large spatial data sets. J R Stat Soc Series B Stat Methodol. 2008;70:825–48. 10.1111/j.1467-9868.2008.00663.x PubMed https://doi.org/10.1111/j.1467-9868.2008.00663.x</jrn>

<eref>3. GADM database of Global Administrative Areas, version 3.6 [cited 2020 Feb 14]. https://biogeo.ucdavis.edu/data/gadm3.6/gpkg/gadm36_GBR_gpkg.zip.</eref>

Appendix Table 1. Regular expression used to screen for cases of frequent vomiting in the clinical free text of EHRs, including examples of true positive and false positive patterns it matches. Bold text identifies the precise text string matched by the regular expression.

<table>
<tr><th>Category</th><th>Code/description</th></tr>
<tr><td>Regular expression</td><td>(?:(?:\W(?:[3-9]\W?x|severe|profuse|prolific|non[\-\s]stop|frequent))\W?(?<!no)(?<!no\ssign\sof)(?<!not)(?<!no\Wmore)(?<!stopped)\W?(?:v[oi]?m+i?t?t?(?:ing|ed)?|v\+{1,10}|(?:has\Wbeen|was)\Wsick)\W(?!stopped)\W?)|(?:(?<!no)(?<!no\ssign\sof)(?<!not)(?<!no\Wmore)(?<!stopped)\W?(?:v[oi]?m+i?t?t?(?:ing|ed)?|v\+{1,10}|(?:has\Wbeen|was)\Wsick)\W(?!stopped)\W?\W?(?:frequently|profusely|(?:(?:[3-9]|\d\d?|(\d\d?\W?\-\W?\d\d?)|many|lots\Wof)\W?(?:times|x)|x\W?(?:[3-9]|\d\d)(?:x|times)?)))</td></tr>
<tr><td>Examples of matching text (bold text) that appear to match profuse vomiting definition</td><td>OR V+ 3 times over last 24h
OR vomited 7 times since this lunch time
vomited 5 times today
profuse vomiting o'night , no diarrhoea empty abdo
<<identifier>> has been sick 2-3 times this afternoon
Has been vomiting frequently today</td></tr>
<tr><td>Example of a false positive matches</td><td>Booster tricat/fel<u>v+ 6 x</u> endectrid</td></tr>
</table>

Appendix Table 2. Descriptive findings of veterinary professional-provided control questionnaire responses, seeking to gain location, signalment, feeding and contact information from dogs that have not recently been observed to prolifically vomit (*n*=60).

Variables	% of responses (95% CI)	n unknown
Practice location		
England	83.3 (73.8–92.8)	NA
Wales	6.7 (0.3–13.0)	NA
Scotland	6.7 (0.3–13.0)	NA
North Ireland	3.3 (0.0–7.9)	NA
SAVSNET-participating practice	14.7 (2.6–26.8)	26
Sex		
F	58.3 (45.7-70.9)	0
M	41.7 (29.1–54.3)	0
Neutered‡	78.3 (67.8–88.9)	0
Intact‡	21.7 (11.1-32.2)	0
Lives in multidog household	51.7 (38.9–64.4)	0
Additional dog in household vomited	32.3 (15.5–49.0)	29
Vaccinated within past 3 years†	95.0 (89.4–100.6)	0
Distemper	93.3 (87.0–99.7)	NA
Infectious hepatitis	93.3 (87.0–99.7)	NA
Parvo	91.7 (84.6–98.7)	NA
Parainfluenza	56.7 (44.0–69.3)	NA
Leptospirosis	93.3 (87.0–99.7)	NA
Kennel cough	48.3 (35.6–61.1)	NA
Rabies	10.0 (2.3–17.7)	NA
Dewormed within previous 3 months	84.2 (74.7–93.8)	3
Other species regular contact†	66.0 (53.2–78.9)	7
Cats	74.3 (59.6–89.0)	NA
Horses	25.7 (11.0–40.4)	NA
Cattle and/or sheep	22.9 (8.7–37.0)	NA
Pigs	2.9 (0.0–8.5)	NA
Poultry	22.9 (8.7–37.0)	NA
Other species	14.3 (2.5–26.1)	NA
Recent travel history†	32.1 (19.4–44.8)	7
Boarding kennel	5.9 (0.0–17.4)	NA
Group training/behavior classes	35.3 (11.9–58.7)	NA
Dog day care facility	17.7 (0.0–36.3)	NA
Overseas	5.9 (0.0–17.4)	NA
Rescue kennel	0.0 (0.0–0.0)	NA
Other	47.1 (22.6–71.5)	NA
Provided food type known†	95.0 (89.4–100.6)	0
Proprietary dog food	89.5 (81.4–97.5)	NA
Home-cooked diet	3.5 (0.0–8.3)	NA
Raw meat	10.5 (2.5–18.6)	NA
Table scraps	14.0 (4.9–23.1)	NA
Dog scavenges food	23.6 (12.3–35.0)	5
Contact with other vomiting species	30.6 (17.6–43.7)	11

*NA, information not available.
†Multiple responses for the same dog are possible.
‡Both female and male dogs.

Appendix Table 3: Univariable findings from logistic regression model exploring the odds of being a veterinary professional-reported prolific vomiting case against a set of veterinary professional-provided control dogs*

Variable	β	SE	OR (95% CI)	p value
Veterinary location, country				
England†	1.02	0.20	1.00	NA
Northern Ireland or ROI	–0.32	0.92	0.73 (0.12–4.41)	0.73
Scotland	–0.30	0.66	0.74 (0.20–2.68)	0.65
Wales	0.63	0.59	1.88 (0.59–5.93)	0.28
Sex				
F†	0.73	0.23	1.00	NA
M	0.71	0.33	2.02 (1.06–3.86)	0.03
Neutered status				
Not neutered†	1.42	0.33	1.00	NA
Neutered	–0.49	0.36	0.62 (0.30–1.26)	0.18
Sex and neutered status				
Neutered F†	0.60	0.26	1.00	NA
F, intact	0.48	0.50	1.61 (0.60–4.29)	0.34
M, intact	1.25	0.57	3.47 (1.14–10.55)	0.03
Neutered M	0.70	0.38	2.01 (0.95–4.23)	0.07
No. dogs in household				
Single dog household†	1.36	0.24	1.00	NA
Multidog household	–0.72	0.32	0.49 (0.26–0.90)	0.02
No. dogs vomiting in multidog household				
0†	0.24	0.31	1.00	NA
One or more	0.93	0.48	2.52 (0.99–6.43)	0.05
Single dog household	1.11	0.37	3.04 (1.48–6.27)	<0.01
Vaccination status				
Not recently vaccinated†	1.13	0.69	1.00	NA
Recently vaccinated	–0.07	0.70	0.93 (0.23–3.70)	0.92
Deworming status				
Not recently dewormed†	0.76	0.42	1.00	NA
Recently dewormed	0.21	0.46	1.23 (0.50–3.06)	0.65
Unknown	1.55	0.76	4.73 (1.06–21.16)	0.04
Contact with other species				
No†	1.17	0.30	1.00	NA
Yes	–0.48	0.36	0.62 (0.31–1.24)	0.17
Unknown	0.74	0.51	2.09 (0.77–5.66)	0.15
Contact with cats				
No contact†	1.14	0.26	1.00	NA
Contact	–0.61	0.35	0.55 (0.27–1.09)	0.09
Unknown	0.78	0.48	2.17 (0.84–5.61)	0.11
Contact with horses				
No contact†	0.95	0.21	1.00	NA
Contact	–0.48	0.48	0.62 (0.24–1.61)	0.33
Unknown	0.96	0.47	2.62 (1.05–6.52)	0.04
Contact with cattle and/or sheep				
No contact†	0.90	0.20	1.00	NA
Contact	–0.11	0.49	0.90 (0.35–2.33)	0.83
Unknown	1.01	0.47	2.76 (1.11–6.87)	0.03
Contact with pigs				
No contact†	0.88	0.19	1.00	NA
Contact	–0.14	1.30	0.87 (0.07–11.06)	0.91
Unknown	1.03	0.46	2.79 (1.13–6.89)	0.03
Contact with poultry				
No contact†	0.99	0.21	1.00	NA
Contact	–0.90	0.56	0.41 (0.14–1.22)	0.11
Unknown	0.95	0.47	2.58 (1.03–6.43)	0.04
Contact with other species				
No contact†	0.88	0.19	1.00	NA
Contact	0.02	0.60	1.02 (0.32–3.31)	0.97
Unknown	1.03	0.47	2.81 (1.13–6.99)	0.03
Dog travel status				
No recent travel†	0.84	0.22	1.00	NA
Recent travel	–0.03	0.36	0.97 (0.48–1.97)	0.93
Unknown	1.10	0.46	3.01 (1.22–7.40)	0.02
Travel to boarding kennel				
No travel†	0.82	0.19	1.00	NA
Travel	0.29	1.19	1.34 (0.13–13.70)	0.81
Unknown	1.12	0.45	3.06 (1.28–7.32)	0.01

Variable	β	SE	OR (95% CI)	p value
Travel to training class				
No travel†	0.87	0.20	1.00	NA
Travel	–0.45	0.57	0.64 (0.21–1.95)	0.43
Unknown	1.07	0.45	2.91 (1.21–7.01)	0.02
Travel to dog day care				
No travel†	0.73	0.19	1.00	NA
Travel	1.14	0.66	3.12 (0.85–11.44)	0.09
Unknown	1.23	0.45	3.41 (1.41–8.25)	0.01
Overseas travel				
No travel†	0.84	0.19	1.00	NA
Travel	–0.84	1.46	0.43 (0.03–7.55)	0.57
Unknown	1.10	0.45	3.01 (1.26–7.20)	0.01
Other types of travel				
No travel†	0.95	0.21	1.00	NA
Travel	–1.08	0.57	0.34 (0.11–1.04)	0.06
Unknown	1.01	0.45	2.74 (1.13–6.61)	0.03
Food types				
Food types not known†	0.99	0.70	1.00	NA
Food types known	0.07	0.72	1.08 (0.26–4.40)	0.92
Proprietary dog food provided				
Not provided†	0.18	0.58	1.00	NA
Provided	0.95	0.60	2.59 (0.79–8.43)	0.12
Unknown	0.80	0.90	2.23 (0.38–13.06)	0.37
Raw food provided				
Not provided†	1.13	0.20	1.00	NA
Provided	–0.81	0.59	0.45 (0.14–1.40)	0.17
Unknown	–0.14	0.72	0.87 (0.21–3.58)	0.85
Food scraps provided				
Not provided†	1.06	0.20	1.00	NA
Provided	0.06	0.46	1.06 (0.43–2.59)	0.90
Unknown	–0.07	0.72	0.94 (0.23–3.86)	0.93
Dog food scavenger status				
Not a scavenger†	0.81	0.21	1.00	NA
Scavenger	0.62	0.37	1.86 (0.91–3.81)	0.09
Unknown	0.59	0.54	1.80 (0.62–5.23)	0.28
Other species vomiting contact				
No contact†	1.09	0.23	1.00	NA
Contact	–1.08	0.44	0.34 (0.15–0.80)	0.01
Unknown	0.55	0.40	1.74 (0.80–3.78)	0.16
No. dogs in household				
1†	1.29	0.23	1.00	NA
2	–0.58	0.36	0.56 (0.27–1.14)	0.11
3	–0.45	0.59	0.64 (0.20–2.05)	0.45
4	–0.61	0.76	0.54 (0.12–2.43)	0.42
≥5	–0.77	0.79	0.46 (0.10–2.17)	0.33
Age, y				
At time of illness†	2.24	0.58	1.00	NA
Linear term	–0.48	0.28	0.62 (0.36–1.08)	0.09
Quadratic term	0.07	0.04	1.08 (1.00–1.16)	0.06
Cubic term	0.00	0.00	1.00 (0.99–1.00)	0.04

* β, β-value (coefficient); NA, information not available
†Intercept

CASE QUESTIONNAIRE INFORMATION SHEET AND CONSENT FORM

Potential Outbreak Investigation: Prolific Vomiting in Dogs

You are being invited to participate in an outbreak investigation study, following reports of an outbreak of prolific vomiting in dogs. Before you decide whether to participate, it is important for you to understand why the survey is being conducted and what it will involve if you do choose to take part. Please consider the following information. Epidemiologist contact details are listed below should you have any further questions.

Reading this information sheet and completing the survey will be considered as consent to participate in this survey.

What is the purpose of the survey?

This survey has been created in order to collect more detailed case information, following veterinary surgeon and social media reports of a potential outbreak of prolific, acute vomiting in dogs during December 2019 and January 2020.

Why am I being invited to take part and what will happen if I take part?

You are being invited to take part because you are a veterinary surgeon or owner currently working in a companion animal-treating veterinary practice or an owner, in the United Kingdom, who has potentially identified a case fitting the case definition of "dog with acute onset of prolific vomiting, with 5 or more episodes of vomiting within a 12 hour period".

If you decide to take part you will need to complete the online survey, which will take around 10 minutes.

Participation is voluntary and you do not have to take part in this study. You are free to withdraw at any time until you have selected the ‘finish’ button on the final page of the questionnaire. You do not have to give a reason if you do not wish to take part.

If you are willing, we will also request your postcode, name and email address so that we can ask for further case details if this becomes necessary during the potential outbreak investigation. We will only use your name and email for the purpose of seeking further information, and will destroy data containing these personal identifiers on conclusion of the survey.

Are there any benefits or risks in taking part?

There are no direct benefits or risks to you or your practice associated with taking part in this survey, but we will use the data to further characterise this potential outbreak, and if necessary assist in controlling the potential outbreak.

What will happen if I want to stop taking part?

If you want to stop taking part in this survey you can withdraw at any time until completion and submission of the online survey.

How will my data be used?

The data you provide will be stored securely for up to 7 years in line with data protection requirements at the University of Liverpool and GDPR. All data is strictly confidential and only researchers involved in the study will have access to it. Fully anonymised data may be archived for use in other research projects in the future. Under UK data protection legislation, the University acts as the Data Controller for personal data collected as part of the University's research. The Principal Investigator acts as the Data Processor for this study.

What will happen to the results of the survey?

The data will be used to further characterise the potential outbreak of prolific vomiting in dogs, potentially assisting in identifying causative factors and informing attempts (if necessary) to control this potential outbreak. Anonymised results may also be published - you and your clients (if relevant) will never be identifiable.

What if I am unhappy or if there is a problem?

If you are unhappy, or if there is a problem, please feel free to contact the epidemiologists listed below and we will try to help. If you remain unhappy or have a complaint which you feel you cannot communicate directly to the researcher then you should contact the Research Ethics and Integrity Office on 0151 794 8290 (ethics@liv.ac.uk). When contacting the Research Governance Officer, please provide details of the name or description of the study (so that it can be identified), the researcher involved, and the details of the complaint you wish to make.

Dr David Singleton

Dr Gina Pinchbeck

University of Liverpool

Leahurst Campus

Chester High Road

CH64 7TE

Email: savsnet@liverpool.ac.uk

1. Please confirm that you have read and understood the above information and confirm your consent for data to be used for these purposes, as the owner or on behalf of the owner.

- I confirm that I have consent from the owner to collect and submit these data, and I understand that anonymised data may be used in publications

- I confirm that I am the dog's owner, give consent for collection and submission of these data, and I understand that anonymised data may be used in publications

Basic Case information

We are firstly going to ask some basic information pertaining to the case of canine prolific vomiting you would like to report.

2. Are you describing a current or retrospective vomiting case?

- Current (dog vomited 12 hours or less before completion of survey)
- Retrospective (dog last vomited over 12 hours ago)
- Don't know

2a. If describing a retrospective case, please state date of onset of vomiting:

- Free text response box

Current cases:

3. In the last 12 hours before completion of this survey, how many times has the dog vomited?

- Less than five times
- Five times or more*

Retrospective cases:

4. When the dog was vomiting most frequently, approximately how many times did the dog vomit over a 12 hour period?

- Less than five times
- Five times or more*

* Only participants who selected 'five times or more' in questions 3 or 4 (hence describing a case fitting the case definition) were able to proceed with answering the remaining questions in this survey.

5. Which of the following statements best describes yourself:

- I am a veterinary surgeon wishing to report a potential case of prolific vomiting in a dog under my care (1)
- I am an employee of a veterinary practice wishing to report a potential case of prolific vomiting in a dog (2)
- I am a dog owner / main keeper wishing to report a potential prolific vomiting case in my own dog (3)
- Other (4)*

5a. If you selected Other, please specify:

- Free text response box

* Only participants selecting 'Other' in Question 5 were able to answer Question 5a.

Participants selecting options (1) and (2) on question 5 were routed towards the ‘Veterinary Professional Questionnaire’, whereas those selecting (3) and (4) were routed towards the ‘Owner Questionnaire’. These two sub-questionnaires are outlined on the following pages.

Veterinary Professional Questionnaire

Practice: Case Details

This section will ask more details about the dog and veterinary practice under which (s)he is registered.

1. Please provide the name of the veterinary practice under which the dog is registered:

- Free text response box

2. Please provide the postcode of the veterinary practice under which the dog is registered:

- Free text response box

3. Please provide the phone number of the veterinary practice under which the dog is registered:

- Free text response box

4. Please provide the email address of the veterinary practice under which the dog is registered:

- Free text response box

5. Does the veterinary practice in which the dog is registered currently participate in the Small Animal Veterinary Surveillance Network (SAVSNET)?

- Yes
- No
- Don’t know

Dog

6. Please provide the name of the dog:

- Free text response box

7. Please provide the postcode of the dog's owner / main keeper:

- Free text response box

8. Please provide the dog's sex:

- Male
- Female
- Don’t know

9. Is the dog neutered?

- Yes
- No
- Don’t know

10. Please state the dog's age. If unknown, please state 'unknown':

- Free text response box

11. Please state the dog's breed. If unknown, state 'unknown'; if crossbreed, state 'crossbreed'.

- Free text response box

12. Are there any other dogs in the case's household?

- Yes*
- No
- Don’t know

*Only participants selecting ‘Yes’ on Question 12 were able to answer questions 12a and 12b.

12a. INCLUDING this dog, how many dogs are there in the household?

- Free text response box

12b. Since onset of vomiting, have any other dogs exhibited signs of vomiting?

- Yes
- No
- Don’t know

13. Has the dog been vaccinated within the last three years?

- Yes*
- No
- Don’t know

* Only participants selecting ‘Yes’ on Question 13 were able to answer questions 13a, 13b (if relevant) and 13c.

13a. Please tick which of the following infectious diseases the dog has been inoculated against (please tick all that apply):

- Distemper
- Infectious hepatitis
- Parvo
- Parainfluenza
- Leptospirosis
- Kennel cough
- Don't know
- Other*

* Only participants selecting ‘Other’ in Question 13a were able to answer Question 13b.

13b. If you selected Other, please specify:

- Free text response box

13c. If known, please state which brand(s) of vaccine have been used at the LAST vaccination/booster of this dog:

- Free text response box

14. Has the dog been de-wormed within the last three months?

- Yes*
- No
- Don’t know

* Only participants selecting ‘Yes’ on Question 14 were able to answer questions 14a.

14a. Which de-worming product was used?

- Free text response box

15. Are there any other animal species which the dog has regular contact (either directly, or with their faeces)? Please tick all that apply.

- None
- Cats
- Pigs
- Cattle / sheep
- Horses
- Poultry
- Don't know

- Other*

* Only participants selecting ‘Other’ on Question 15 were able to answer questions 15a.

15a. If you selected Other, please specify:

- Free text response box

16. In the last month, has the dog been to any of the following (please tick all that apply):

- None
- Boarding kennel
- Rescue kennel
- Overseas
- Dog day care facility
- Group training / behaviour classes
- Don't know
- Other*

* Only participants selecting ‘Other’ on Question 16 were able to answer questions 16a.

16a. If you selected Other, please specify:

- Free text response box

17. Which of the following food types does the dog regularly eat?

- Proprietary dog food
- Home-cooked diet
- Raw meat
- Table scraps
- Don't know
- Other*

* Only participants selecting ‘Other’ on Question 17 were able to answer questions 17a.

17a. If you selected Other, please specify:

- Free text response box

18. Does the dog scavenge food (e.g. from bins when out walking)?

- Yes
- No
- Don’t know

19. In the seven days prior to onset of vomiting, did the dog have any contact with other animals or humans that had been vomiting?

- Yes
- No
- Don’t know

20. Which clinical signs has this dog exhibited (please tick all that apply)?

- Vomiting without blood
- Vomiting with blood
- Diarrhoea without blood
- Diarrhoea with blood
- Melaena
- Weight loss
- Inappetence
- Pyrexia
- Other*

* Only participants selecting ‘Other’ on Question 20 were able to answer questions 20a.

20a. If you selected Other, please specify:

- Free text response box

21. At time of latest examination, if recorded please state the body temperature of the dog (in Celsius):

- Free text response box

22. Was any treatment prescribed for this dog?

- Yes*
- No
- Don’t know

* Only participants selecting ‘Yes’ on Question 22 were able to answer questions 22a.

22a. If known, please state which treatments were provided:

- Free text response box

23. Were any samples taken, or diagnostic tests performed?

- Yes*
- No
- Don’t know

*** Only participants selecting ‘Yes’ on Question 23 were able to answer questions 23a.**

23a. Please state which samples were taken and which diagnostic tests were performed. If you know the result(s) of such diagnostic tests, please also state this here:

- Free text response box

24. How long did the dog take to recover?

- Less than 24 hours
- 24 - 48 hours
- 3 - 7 days
- 8 - 14 days
- More than 14 days
- Dog is still vomiting
- Dog has stopped vomiting, but is still unwell
- Dog died
- Don't know

25. Please provide ANY OTHER relevant information about this dog.

- Free text response box

Practice: Control Cases

When investigating a potential disease outbreak, it is important to collect information relating to a population of animals NOT exhibiting clinical signs associated with the outbreak under investigation (the 'control population'). If possible, please complete some further questions relating to **a randomly selected dog NOT exhibiting vomiting clinical signs** that presented at your veterinary practice **on the same day** the affected animal presented e.g. the next non-vomiting dog you see where the owner is happy to participate.

1. Please confirm that you are able, and willing, to provide information regarding a control dog that has not reported to the veterinary practice with vomiting clinical signs within the last month.

- I am willing and able to provide information on a non-vomiting control dog*
- I am NOT willing or able to provide information on a non-vomiting control dog

* Only participants who selected ‘I am willing and able to provide information on a non-vomiting control dog’ in Question 1 were able to proceed with answering the questions

pertaining to a control animal in this survey. Those who were not willing were directed to submit the case questionnaire details they had provided alone.

Practice: Control Details

1. Has the chosen control dog presented to the veterinary practice with vomiting clinical signs within the last month?

- Yes - please select another dog

- No*

- Don't know - please select another dog

* Only participants who selected ‘Yes’ in Question 1 were able to proceed with answering the questions pertaining to a control animal in this survey. Those who were not willing were directed to submit the case questionnaire details they had provided alone.

Veterinary Practice

1. Please provide the name of the veterinary practice under which the dog is registered:

- Free text response box

2. Please provide the postcode of the veterinary practice under which the dog is registered:

- Free text response box

3. Does the veterinary practice in which the dog is registered currently participate in the Small Animal Veterinary Surveillance Network (SAVSNET)?

- Yes

- No

- Don’t know

Dog

4. Please provide the name of the dog:

- Free text response box

5. Please provide the postcode of the dog's owner / main keeper:

- Free text response box

6. Please provide the dog's sex:

- Male
- Female
- Don’t know

7. Is the dog neutered?

- Yes
- No
- Don’t know

8. Please state the dog's age. If unknown, please state 'unknown':

- Free text response box

9. Please state the dog's breed. If unknown, state 'unknown'; if crossbreed, state 'crossbreed'.

- Free text response box

10. Are there any other dogs in the case's household?

- Yes*
- No
- Don’t know

*Only participants selecting ‘Yes’ on Question 10 were able to answer questions 10a and 10b.

10a. INCLUDING this dog, how many dogs are there in the household?

- Free text response box

10b. Have any other dogs exhibited signs of vomiting?

- Yes
- No
- Don’t know

11. Has the dog been vaccinated within the last three years?

- Yes*
- No
- Don’t know

* Only participants selecting ‘Yes’ on Question 11 were able to answer questions 11a, 11b (if relevant) and 11c.

11a. Please tick which of the following infectious diseases the dog has been inoculated against (please tick all that apply):

- Distemper
- Infectious hepatitis
- Parvo
- Parainfluenza
- Leptospirosis
- Kennel cough
- Don't know
- Other*

* Only participants selecting ‘Other’ in Question 11a were able to answer Question 11b.

11b. If you selected Other, please specify:

- Free text response box

11c. If known, please state which brand(s) of vaccine have been used at the LAST vaccination/booster of this dog:

- Free text response box

12. Has the dog been de-wormed within the last three months?

- Yes*
- No
- Don’t know

* Only participants selecting ‘Yes’ on Question 12 were able to answer questions 12a.

12a. Which de-worming product was used?

- Free text response box

13. Are there any other animal species which the dog has regular contact (either directly, or with their faeces)? Please tick all that apply.

- None
- Cats
- Pigs
- Cattle / sheep
- Horses
- Poultry
- Don't know

- Other*

* Only participants selecting ‘Other’ on Question 13 were able to answer questions 13a.

13a. If you selected Other, please specify:

- Free text response box

14. In the last month, has the dog been to any of the following (please tick all that apply):

- None
- Boarding kennel
- Rescue kennel
- Overseas
- Dog day care facility
- Group training / behaviour classes
- Don't know
- Other*

* Only participants selecting ‘Other’ on Question 14 were able to answer questions 14a.

14a. If you selected Other, please specify:

- Free text response box

15. Which of the following food types does the dog regularly eat?

- Proprietary dog food
- Home-cooked diet
- Raw meat
- Table scraps
- Don't know
- Other* * Only participants selecting ‘Other’ on Question 15 were able to answer questions 15a.

15a. If you selected Other, please specify:

- Free text response box

16. Does the dog scavenge food (e.g. from bins when out walking)?

- Yes
- No
- Don’t know

17. Has the dog had any contact with other animals or humans that had been vomiting?

- Yes
- No
- Don’t know

OWNER QUESTIONNAIRE

Owner: Case Details

This section will ask more details about the dog and veterinary practice under which (s)he is registered.

1. Please provide the name of the veterinary practice under which the dog is registered:

- Free text response box

2. Please provide the postcode of the veterinary practice under which the dog is registered:

- Free text response box

3. Does the veterinary practice in which the dog is registered currently participate in the Small Animal Veterinary Surveillance Network (SAVSNET)?

- Yes
- No
- Don’t know

Dog

4. Please provide the name of the dog:

- Free text response box

5. Please provide the postcode of the dog's owner / main keeper:

- Free text response box

6. Please provide the dog's sex:

- Male
- Female
- Don’t know

7. Is the dog neutered?

- Yes
- No
- Don’t know

8. Please state the dog's age. If unknown, please state 'unknown':

- Free text response box

9. Please state the dog's breed. If unknown, state 'unknown'; if crossbreed, state 'crossbreed'.

- Free text response box

10. Are there any other dogs in the case's household?

- Yes*
- No
- Don’t know

*Only participants selecting ‘Yes’ on Question 12 were able to answer questions 12a and 12b.

10a. INCLUDING this dog, how many dogs are there in the household?

- Free text response box

10b. Since onset of vomiting, have any other dogs exhibited signs of vomiting?

- Yes
- No
- Don’t know

11. Has the dog been vaccinated within the last three years?

- Yes*
- No
- Don’t know

* Only participants selecting ‘Yes’ on Question 11 were able to answer questions 11a, 11b (if relevant) and 13c.

11a. Please tick which of the following infectious diseases the dog has been inoculated against (please tick all that apply):

- Distemper
- Infectious hepatitis
- Parvo

- Parainfluenza
- Leptospirosis
- Kennel cough
- Don't know
- Other*

* Only participants selecting ‘Other’ in Question 11a were able to answer Question 11b.

11b. If you selected Other, please specify:

- Free text response box

11c. If known, please state which brand(s) of vaccine have been used at the LAST vaccination/booster of this dog:

- Free text response box

12. Has the dog been de-wormed within the last three months?

- Yes*
- No
- Don’t know

* Only participants selecting ‘Yes’ on Question 12 were able to answer questions 12a.

12a. Which de-worming product was used?

- Free text response box

13. Are there any other animal species which the dog has regular contact (either directly, or with their faeces)? Please tick all that apply.

- None
- Cats
- Pigs
- Cattle / sheep
- Horses
- Poultry
- Don't know
- Other*

* Only participants selecting ‘Other’ on Question 13 were able to answer questions 13a.

13a. If you selected Other, please specify:

- Free text response box

14. In the last month, has the dog been to any of the following (please tick all that apply):

- None
- Boarding kennel
- Rescue kennel
- Overseas
- Dog day care facility
- Group training / behaviour classes
- Don't know
- Other*

* Only participants selecting ‘Other’ on Question 14 were able to answer questions 14a.

14a. If you selected Other, please specify:

- Free text response box

15. Which of the following food types does the dog regularly eat?

- Proprietary dog food
- Home-cooked diet
- Raw meat
- Table scraps
- Don't know
- Other*

* Only participants selecting ‘Other’ on Question 15 were able to answer questions 15a.

15a. If you selected Other, please specify:

- Free text response box

16. Does the dog scavenge food (e.g. from bins when out walking)?

- Yes
- No
- Don’t know

17. In the seven days prior to onset of vomiting, did the dog have any contact with other animals or humans that had been vomiting?

- Yes
- No
- Don’t know

18. Which clinical signs has this dog exhibited (please tick all that apply)?

- Vomiting without blood
- Vomiting with blood
- Diarrhoea without blood
- Diarrhoea with blood
- Weight loss
- Inappetence
- Fever
- Other*

* Only participants selecting ‘Other’ on Question 18 were able to answer questions 18a.

18a. If you selected Other, please specify:

- Free text response box

19. Was any treatment prescribed for this dog?

- Yes*
- No
- Don’t know

* Only participants selecting ‘Yes’ on Question 19 were able to answer questions 19a.

19a. If known, please state which treatments were provided:

- Free text response box

20. Were any samples taken, or diagnostic tests performed?

- Yes*
- No
- Don’t know

*** Only participants selecting ‘Yes’ on Question 20 were able to answer questions 20a.**

20a. Please state which samples were taken and which diagnostic tests were performed. If you know the result(s) of such diagnostic tests, please also state this here:

- Free text response box

21. How long did the dog take to recover?

- Less than 24 hours
- 24 - 48 hours
- 3 - 7 days

- 8 - 14 days
- More than 14 days
- Dog is still vomiting
- Dog has stopped vomiting, but is still unwell
- Dog died
- Don't know

22. Please provide ANY OTHER relevant information about this dog.

- Free text response box

CONTROL QUESTIONNAIRE INFORMATION SHEET AND CONSENT FORM

Potential outbreak investigation: Prolific vomiting in dogs

CONTROL QUESTIONNAIRE

You are being invited to participate in an outbreak investigation study, following reports of an outbreak of prolific vomiting in dogs. Before you decide whether to participate, it is important for you to understand why the survey is being conducted and what it will involve if you do choose to take part. Please consider the following information. Epidemiologist contact details are listed below should you have any further questions.

Reading this information sheet and completing the survey will be considered as consent to participate in this survey.

What is the purpose of the survey?

This survey has been created in order to collect more detailed CONTROL information, following veterinary surgeon and social media reports of a potential outbreak of prolific, acute vomiting in dogs during December 2019 and January 2020.

Why am I being invited to take part and what will happen if I take part?

You are being invited to take part because you are a veterinary surgeon or owner currently working in a companion animal-treating veterinary practice or an owner, in the United Kingdom, who is willing to provide information on CONTROL dogs, as part of an ongoing investigation concerning dogs with acute onset of prolific vomiting, with 5 or more episodes of vomiting within a 12 hour period". If you would like to submit information about a CASE, please click here.

If you decide to take part you will need to complete the online survey, which will take around 10 minutes.

Participation is voluntary and you do not have to take part in this study. You are free to withdraw at any time until you have selected the ‘finish’ button on the final page of the questionnaire. You do not have to give a reason if you do not wish to take part.

If you are willing, we will also request your postcode, name and email address so that we can ask for further CONTROL details if this becomes necessary during the potential outbreak investigation. We will only use your name and email for the purpose of seeking further information, and will destroy data containing these personal identifiers on conclusion of the survey.

Are there any benefits or risks in taking part?

There are no direct benefits or risks to you or your practice associated with taking part in this survey, but we will use the data to further characterise this potential outbreak, and if necessary assist in controlling the potential outbreak.

What will happen if I want to stop taking part?

If you want to stop taking part in this survey you can withdraw at any time until completion and submission of the online survey.

How will my data be used?

The data you provide will be stored securely for up to 7 years in line with data protection requirements at the University of Liverpool and GDPR. All data is strictly confidential and only researchers involved in the study will have access to it. Fully anonymised data may be archived for use in other research projects in the future. Under UK data protection legislation, the University acts as the Data Controller for personal data collected as part of the University’s research. The Principal Investigator acts as the Data Processor for this study.

What will happen to the results of the survey?

The data will be used to further characterise the potential outbreak of prolific vomiting in dogs, potentially assisting in identifying causative factors and informing attempts (if necessary) to control this potential outbreak. Anonymised results may also be published - you and your clients (if relevant) will never be identifiable.

What if I am unhappy or if there is a problem?

If you are unhappy, or if there is a problem, please feel free to contact the epidemiologists listed below and we will try to help. If you remain unhappy or have a complaint which you feel you cannot communicate directly to the researcher then you should contact the Research Ethics and Integrity Office on 0151 794 8290 (ethics@liv.ac.uk). When contacting the Research Governance Officer, please provide details of the name or description of the study (so that it can be identified), the researcher involved, and the details of the complaint you wish to make.

Dr David Singleton

Dr Gina Pinchbeck
University of Liverpool
Leahurst Campus
Chester High Road
CH64 7TE
Email: savsnet@liverpool.ac.uk

Please confirm that you have read and understood the above information and confirm your consent for data to be used for these purposes, as the owner or on behalf of the owner.

- I confirm that I have consent from the owner to collect and submit these data, and I understand that anonymised data may be used in publications
- I confirm that I am the dog's owner, give consent for collection and submission of these data, and I understand that anonymised data may be used in publications

---

Basic CONTROL information

1. Which of the following statements best describes yourself:

- I am a veterinary surgeon wishing to provide information about a control dog
- I am an employee of a veterinary practice wishing to provide information about a control dog
- I am a dog owner / main keeper wishing to provide information about a control dog
- Other

* Only participants selecting ‘Other’ on Question 1 were able to answer questions 1a.

1a. If you selected Other, please specify:

- Free text response box

Control Cases

When investigating a potential disease outbreak, it is important to collect information relating to a population of animals **NOT** exhibiting clinical signs associated with the outbreak under investigation (the 'control population'). If possible, please complete some further questions relating to **a randomly selected dog NOT exhibiting vomiting clinical signs.**

2. Please confirm that you are able, and willing, to provide information regarding a CONTROL dog that has NOT exhibited vomiting clinical signs within the last month.

- I am willing and able to provide information on a non-vomiting control dog*

- I am NOT willing or able to provide information on a non-vomiting control dog

* Only participants who selected ‘I am willing and able to provide information on a non-vomiting control dog’ in Question 2 were able to proceed with answering the questions pertaining to a control animal in this survey.

Control details

Veterinary Practice

3. Please provide the name of the veterinary practice under which the dog is registered:

- Free text response box

4. Please provide the postcode of the veterinary practice under which the dog is registered:

- Free text response box

5. Does the veterinary practice in which the dog is registered currently participate in the Small Animal Veterinary Surveillance Network (SAVSNET)?

- Yes
- No
- Don’t know

Dog

6. Please provide the name of the dog:

- Free text response box

7. Please provide the postcode of the dog's owner / main keeper:

- Free text response box

8. Please provide the dog's sex:

- Male
- Female
- Don’t know

9. Is the dog neutered?

- Yes
- No
- Don’t know

10. Please state the dog's age. If unknown, please state 'unknown':

- Free text response box

11. Please state the dog's breed. If unknown, state 'unknown'; if crossbreed, state 'crossbreed'.

- Free text response box

12. Are there any other dogs in the case's household?

- Yes*
- No
- Don’t know

*Only participants selecting ‘Yes’ on Question 12 were able to answer questions 12a and 12b.

12a. INCLUDING this dog, how many dogs are there in the household?

- Free text response box

12b. Have any other dogs exhibited signs of vomiting?

- Yes
- No
- Don’t know

13. Has the dog been vaccinated within the last three years?

- Yes*
- No
- Don’t know

* Only participants selecting ‘Yes’ on Question 13 were able to answer questions 13a, 13b (if relevant) and 13c.

13a. Please tick which of the following infectious diseases the dog has been inoculated against (please tick all that apply):

- Distemper
- Infectious hepatitis
- Parvo
- Parainfluenza
- Leptospirosis
- Kennel cough
- Don't know
- Other*

* Only participants selecting ‘Other’ in Question 13a were able to answer Question 13b.

13b. If you selected Other, please specify:

- Free text response box

13c. If known, please state which brand(s) of vaccine have been used at the LAST vaccination/booster of this dog:

- Free text response box

14. Has the dog been de-wormed within the last three months?

- Yes*
- No
- Don’t know

* Only participants selecting ‘Yes’ on Question 14 were able to answer questions 14a.

14a. Which de-worming product was used?

- Free text response box

15. Are there any other animal species which the dog has regular contact (either directly, or with their faeces)? Please tick all that apply.

- None
- Cats
- Pigs
- Cattle / sheep
- Horses
- Poultry
- Don't know
- Other*

* Only participants selecting ‘Other’ on Question 15 were able to answer questions 15a.

15a. If you selected Other, please specify:

- Free text response box

16. In the last month, has the dog been to any of the following (please tick all that apply):

- None
- Boarding kennel
- Rescue kennel

- Overseas
- Dog day care facility
- Group training / behaviour classes
- Don't know
- Other*

* Only participants selecting ‘Other’ on Question 16 were able to answer questions 16a.

16a. If you selected Other, please specify:

- Free text response box

17. Which of the following food types does the dog regularly eat?

- Proprietary dog food
- Home-cooked diet
- Raw meat
- Table scraps
- Don't know
- Other*

* Only participants selecting ‘Other’ on Question 17 were able to answer questions 17a.

17a. If you selected Other, please specify:

- Free text response box

18. Does the dog scavenge food (e.g. from bins when out walking)?

- Yes
- No
- Don’t know

19. Has the dog had any contact with other animals or humans that had been vomiting?

- Yes
- No
- Don’t know
